# Oridonin induces apoptosis and senescence by increasing hydrogen peroxide and glutathione depletion in colorectal cancer cells

**DOI:** 10.3892/ijmm.2012.895

**Published:** 2012-01-24

**Authors:** FENG-HOU GAO, FENG LIU, WEI WEI, LI-BIN LIU, MANG-HUA XU, ZHU-YING GUO, WEI LI, BIN JIANG, YING-LI WU

**Affiliations:** 1 Number 3 People’s Hospital, Affiliated to Shanghai Jiao-Tong University School of Medicine (SJTU-SM), Shanghai 201900; 2 Department of Pathophysiology and Chemical Biology, Division of Shanghai Universities E-Institutes, Key Laboratory of Cell Differentiation and Apoptosis of National Ministry of Education, Shanghai Jiao-Tong University School of Medicine (SJTU-SM), Shanghai 200025; 3 Zhejiang Provincial Key Laboratory of Medical Genetics, School of Life Sciences, Wenzhou Medical College, Wenzhou, Zhejiang, P.R. China

**Keywords:** oridonin, colorectal cancer, hydrogen peroxide, apoptosis, senescence

## Abstract

We recently demonstrated that oridonin could induce apoptosis and senescence of colon cancer cells *in vitro* and *in vivo*. However, the underlying mechanism remains unknown. In this study, the involvement of reactive oxygen species in oridonin-induced cell death and senescence was investigated in colon adenocarcinoma-derived SW1116 cells. Oridonin increased intracellular hydrogen peroxide levels and reduced the glutathione content in a dose-dependent manner. N-acetylcysteine, a reactive oxygen species scavenger, not only blocked the oridonin-induced increase in hydrogen peroxide and glutathione depletion, but also blocked apoptosis and senescence induced by oridonin, as evidenced by the decrease in Annexin V and senescence-associated β-galactosidase- positive cells and the inhibition of oridonin-induced upregulation of p53 and p16 and downregulation of c-Myc. Moreover, exogenous catalase could inhibit the increase in hydrogen peroxide and apoptosis induced by oridonin, but not the glutathione depletion and senescence. Furthermore, thioredoxin reductase (TrxR) activity was reduced by oridonin *in vitro* and in cells, which may cause the increase in hydrogen peroxide. In conclusion, the increase in hydrogen peroxide and glutathione depletion account for oridonin-induced apoptosis and senescence in colorectal cancer cells, and TrxR inhibition is involved in this process. Given the importance of TrxR as a novel cancer target in colon cancer, oridonin would be a promising clinical candidate. The mechanism of oridonin-induced inhibition of TrxR warrants further investigation.

## Introduction

Reactive oxygen species (ROS) include hydrogen peroxide, nitric oxide, superoxide anions, hydroxyl radicals and peroxynitrite. A growing body of evidence suggests that ROS within cells act as second messengers in the regulation of many important cellular events, including transcription factor activation, gene expression and cellular proliferation, differentiation and senescence (1,2). An excessive production of ROS frequently leads to serious damage in cells, while mild increases in ROS can induce cellular senescence (3,4). ROS can therefore function as antitumorigenic agents (5–7). Many anticancer therapies, such as the chemotherapeutic agent doxorubicin, etoposide and cisplatin, in addition to radiotherapy and photodynamic therapy, can lead to the generation of superoxide and oxygen radicals within carcinoma cells (8,9). In this sense, the modulation of oxidative stress could be a target of anticancer therapies.

A variety of proteins function as ROS scavengers, such as superoxide dismutase, catalase, glutathione peroxidase, thioredoxin reductase (TrxR), ascorbate, glutathione. Among them, TrxR has been suggested as a new target for anticancer drug development because TrxR is overexpressed in colon, pancreas, lung and other cancers (10–12) and knockdown of TrxR could dramatically reduce tumor growth and metastasis in a lung carcinoma mouse model (13). Developing the novel TrxR targeting compound is promising in the treatment of cancer (14).

Oridonin is an ent-kaurane diterpenoid derived from *Rabdosia rubescens* (15). Studies have shown that oridonin induces apoptosis in a variety of cancer cells including cells from prostate cancer, breast cancer, non-small cell lung cancer, acute leukemia, glioblastoma multiforme and melanoma (16–19). In addition, oridonin can also inhibit cell cycle progression and enhance phagocytosis of apoptotic cells by macrophages (20). However, the mechanisms underlying the antitumor activity of oridonin are not fully understood. Interestingly, exposure of acute promyelocytic leukemia NB4 cells to oridonin resulted in a significant increase in ROS generation while the ROS scavenger, N-acetylcysteine (NAC), completely protected NB4 cells from oridonin-induced apoptosis (16). These findings indicate that ROS signaling is involved in oridonin-induced apoptosis. Recently, we demonstrated that oridonin could induce potent growth inhibition, apoptosis, and senescence of colorectal cancer cells *in vitro* and *in vivo* (21). However, the exact mechanism of this process remains largely unknown.

In the present study, the role of ROS and thioredoxin reductase in oridonin-induced cell death and senescence in human colorectal cancer (SW1116) cells were investigated.

## Materials and methods

### Cell culture

The colorectal cancer cell line SW1116 was purchased from the Shanghai Institutes for Biological Sciences. The cells were maintained in a humidified room air containing 5% CO_2_ at 37°C and cultured in DMEM medium (Gibco-BRL) supplemented with 10% fetal bovine serum and 1% penicillin-streptomycin (Gibco-BRL). Cells in the logarithmic phase of growth were used in all experiments.

### Reagents

Oridonin (98% purity) provided by Dr Tang Qingjiu (Shanghai Academy of Agricultural Sciences) was dissolved in DMSO (Sigma, St. Louis, MO) at a stock concentration of 10 mg/ml and stored at −20°C. The cell-permeable ROS scavenger NAC was obtained from Sigma and dissolved in sterile H_2_O to a stock concentration of 100 mM. Catalase was obtained from Sigma and dissolved in 50 mM potassium phosphate buffer at 4,733 U/ml. All stock solutions were wrapped in foil and maintained at 4°C or −20°C.

### Detection and measurement of intracellular hydrogen peroxide and superoxide anion concentrations

Two oxidation-sensitive fluorescent probe dyes, 2′,7′-dichlorodihydrofluorescein diacetate (DCF-DA, Invitrogen, Molecular Probes, Eugene, OR) and dihydroethidium (DHE, Invitrogen Molecular Probes), were used to measure the intracellular hydrogen peroxide and superoxide anion concentrations, respectively. DCF-DA is deacetylated intracellularly by nonspecific esterases and is further oxidized by cellular peroxides to the fluorescent compound 2′,7′-dichlorofluorescein. DHE is a fluorogenic probe that detects superoxide anion radicals with high selectivity. DHE is cell-permeable and reacts with superoxide anions to form ethidium, which in turn intercalates deoxyribonucleic acid and exhibits a red fluorescence. Briefly, cells were treated with oridonin in the presence or absence of NAC or catalase for the indicated time periods. After washing with phosphate-buffered saline (PBS), cells were incubated with 20 μM DCF-DA or 5 μM DHE at 37°C for 30 min according to the manufacturer’s instructions. The fluorescence signals were detected by a FACStar flow cytometer (Beckman Coulter). For each sample, 5,000 or 10,000 events were collected. Hydrogen peroxide and superoxide anion levels were expressed in terms of mean fluorescence intensity.

### Detection of intracellular glutathione (GSH)

Cellular GSH levels were analyzed using 5-chloromethylfluorescein diacetate (CMFDA, Invitrogen, Molecular Probes). Cytoplasmic esterases convert nonfluorescent CMFDA to fluorescent 5-chloromethylfluorescein, which can then react with the glutathione. CMFDA is a useful membrane-permeable dye for determining levels of intracellular glutathione (22–25). Briefly, cells were treated with oridonin in the presence or absence of ROS scavengers, or catalase for the indicated time periods. After washing with PBS, the cells were incubated with 5 μM CMFDA at 37°C for 30 min according to the manufacturer’s instructions. CMF fluorescence was detected by the FACStar flow cytometer (Beckman Coulter). For each sample, 5,000 or 10,000 events were collected.

### Annexin V/PI staining

Apoptosis was determined using Annexin V-fluorescein isothiocyanate (FITC) staining and PI labeling. Annexin V can identify the externalization of phosphatidylserine during apoptotic progression and therefore can detect cells in early apoptosis. Briefly, cells were treated with oridonin in the presence or absence of NAC or catalase for the indicated time periods. After washing twice with cold PBS, cells were resuspended in 500 μl of binding buffer [10 mM HEPES/NaOH (pH 7.4), 140 mM NaCl, 2.5 mM CaCl_2_] at a concentration of 1×10^6^ cells/ml. Next, 5 μl of Annexin V-FITC (Pharmingen, San Diego, CA) and 10 μl of 20 μg/ml PI were added to these cells, which were analyzed with a FACStar flow cytometer (Beckman Coulter). Viable cells were negative for both PI and Annexin V, apoptotic cells were positive for Annexin V and negative for PI, and late apoptotic dead cells displayed both high Annexin V and PI labeling. Non-viable cells that had undergone necrosis were positive for PI and negative for Annexin V.

### Cell senescence assay

Senescence-associated β-galactosidase activity was determined with a senescence detection kit (Biovision, Mountain View, CA) using fixed cells (26). The development of a blue color in the cytoplasm was detected and photographed using a Nikon (Nikon Instruments Inc., Lewisville, TX) inverted microscope equipped with a color CCD camera. Four pictures were taken of each well. β-galactosidase-stained cells and unstained cells were counted and used to calculate a final average ratio of the number of stained to unstained cells in each well.

### Western blot analysis

Cells were incubated with oridonin and/or NAC for the indicated time periods. Cells were then washed in PBS and suspended in five volumes of lysis buffer [20 mM HEPES (pH 7.9), 20% glycerol, 200 mM KCl, 0.5 mM EDTA, 0.5% NP-40, 0.5 mM DTT, 1% protease inhibitor cocktail (Sigma)]. The lysates were then collected and stored at −20°C. Protein concentrations in the supernatants were determined by the Bradford method. Supernatant samples containing 20 μg of total protein were resolved on 10–15% SDS-PAGE gels and transferred onto Immobilon-P PVDF membranes (Millipore, MA) by electroblotting; membranes were then probed with anti-PARP, p16, c-Myc and p53 (Santa Cruz Biotechnology, Inc., Santa Cruz, CA) primary antibodies and subsequently with horseradish peroxidase-conjugated secondary antibodies. The membrane blots were developed using the ECL kit (Amersham, Arlington Heights, IL).

### Determination of TrxR activity by the dithionitrobenzene reduction assay

All experiments were performed in 96-well plates. Recombinant active rat TrxR (0.05 units) was incubated with various concentrations of oridonin for 60 min. TrxR activity was assayed by the dithionitrobenzene method in a solution containing 50 mM Tris-HCl (pH 7.5), 200 μM NADPH, 5 mM DTNB, and 1 mM EDTA. The absorbance at 412 nm was measured with a Synergy H4 Hybrid Microplate Reader (Bio-Tek, Winooski, VT). A blank reading without TrxR was subtracted from every sample. TrxR enzyme activity was calculated as a percentage of the activity of the DMSO vehicle-treated sample.

### Determination of TrxR activity in cell lysates

The activity of TrxR in cell lysates was measured using the TrxR assay kit (Cayman Chemical, Ann Arbor, MI) according to the manufacturer’s instructions. Briefly, cells were collected by scraping and were homogenized on ice in cold buffer (50 mM potassium phosphate and 1 mM EDTA, pH 7.4). The protein concentration in the supernatant of the lysate was determined using the Bradford method. Protein samples, NADPH, and DTNB were added to the assay buffer in 96-well plates and gently mixed. The absorbance at 412 nm was measured with a Synergy H4 Hybrid Microplate Reader. A blank reading without protein was subtracted from every sample. TrxR enzyme activity was calculated as a percentage of the activity of the DMSO vehicle-treated control sample.

### Statistical analysis

The Student’s t-test was used to evaluate the differences between two different groups. A P-value <0.05 was considered statistically significant.

## Results

### Oridonin increases hydrogen peroxide and decreases superoxide anion and glutathione levels in SW1116 cells

**Figure 1:**
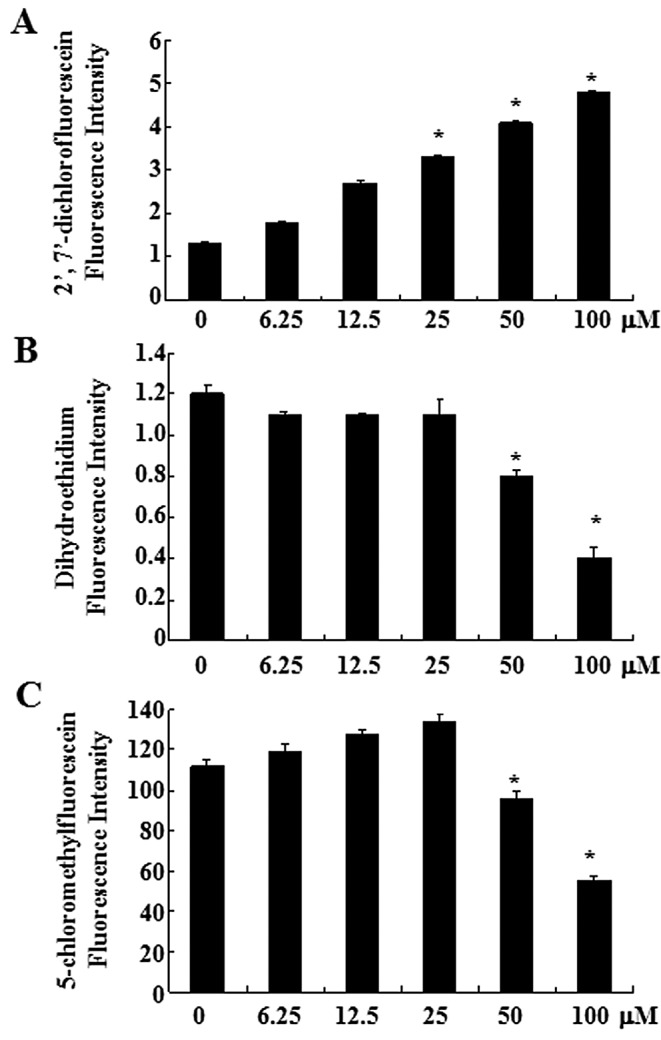
Effects of oridonin (0, 6.25, 12.5, 25, 50 or 100 μm) on intracellular hydrogen peroxide, superoxide anion and glutathione production in SW1116 cells. SW1116 cells were treated with the indicated concentrations of oridonin for 2 h. Intracellular levels of (A) hydrogen peroxide, (B) superoxide anions, and (C) glutathione production were determined by Flow Cytometry. The graphs show the mean fluorescence levels of (A) DCF, (B) DHE and (C) CMF. Each experiment was repeated three times. ^*^P<0.05 when compared with the control group from three independent experiments.

First, the production of intracellular hydrogen peroxide was assessed in oridonin-treated SW1116 cells using the DCF-DA fluorescence dye. As shown in Fig. 1A, oridonin increased the intracellular hydrogen peroxide level in a dose-dependent manner. Treatment with oridonin at 6.25 μM for 2 h increased the intracellular hydrogen peroxide levels, compared with those in the untreated control cells. The intracellular superoxide anion level in oridonin-treated SW1116 cells was assessed using the DHE fluorescence dye. Red fluorescence derived from DHE, which reflects superoxide anion accumulation, decreased significantly in SW1116 cells treated with 50 and 100 μM oridonin, compared with the untreated control cells (Fig. 1B).

As an important component of the antioxidant system, cellular GSH has been shown to be crucial for the regulation of cell proliferation, cell cycle progression, apoptosis and senescence. We therefore analyzed the changes of GSH level in oridonin-treated SW1116 cells by using the CMF fluorescence probe. Oridonin at 50 or 100 μM significantly decreased the level of intracellular GSH content at 2 h (Fig. 1C), indicating the depletion of intracellular GSH content of SW1116 cells. The decrease of CMF fluorescence was observed within 120 min at the exposure to oridonin (50 and 100 μM) (Fig. 1C). However, at a lower concentration (6.25–25 μM), a slight increase of the CMF fluorescence was detected at 2 h.

### The ROS scavenger NAC protects SW1116 cells from oridonin-induced apoptosis or senescence

**Figure 2:**
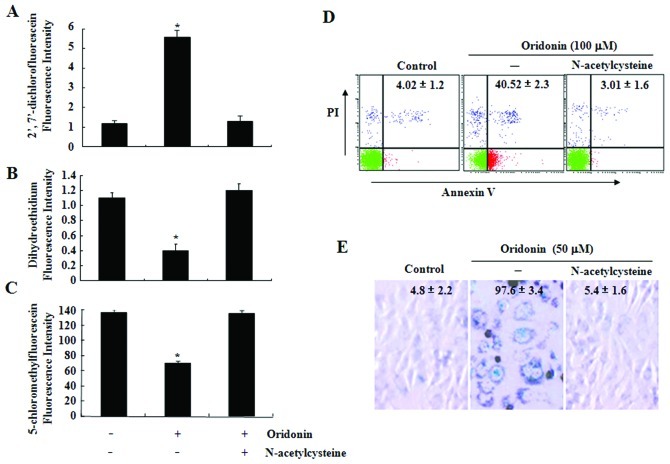
Effects of a ROS scavenger on intracellular ROS and GSH production, oridonin-induced apoptosis and senescence in oridonin-treated SW1116 cells. SW1116 cells were treated with the ROS scavenger NAC and/or oridonin for 2 h. Intracellular (A) hydrogen peroxide, (B) superoxide anion and (C) GSH levels were determined by FACS. The graphs show the mean fluorescence levels of (A) DCF, (B) DHE, and (C) CMF. (D–E) SW1116 cells were treated with NAC and/or oridonin for 12 or 48 h. (D) Annexin-positive cells were measured by flow cytometry. (E) Senescent cells were quantified by senescence-associated β-galactosidase activity analysis (x100). Each experiment was repeated three times. ^*^P<0.05 when compared with the cells untreated or treated with oridonin plus NAC from three independent experiments.

To determine the role of ROS production in oridonin-induced apoptosis and senescence, SW1116 cells were treated with oridonin in the presence or absence of NAC, a well known ROS scavenger, for 2 h. As expected, NAC fully reversed the oridonin-induced increase in hydrogen peroxide as well as the decrease in superoxide anion and GSH levels (Fig. 2A–C). Next, effects of NAC on oridonin-induced cell death and senescence in SW1116 cells were also examined. As shown in Fig. 2D and E, co-treatment with NAC and oridonin completely protected the cells from oridonin-induced cell death and senescence.

### NAC abrogates oridonin-induced changes in poly(ADP-ribose) polymerase (PARP), p16, p53 and c-Myc in SW1116 cells

**Figure 3:**
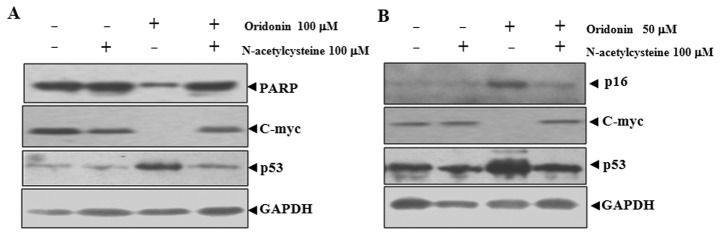
Effects of N-acetylcysteine on oridonin-induced apoptotic and senescence-related proteins in SW1116 cells. SW1116 cells were treated with NAC and/or oridonin for (A) 12 h (100 μM) and (B) 48 h (50 μM). Aliquots of protein extracts (40 μg) were immunoblotted with the indicated antibodies.

Oridonin-induced apoptosis and senescence were related to the upregulation of p53 and p16 and downregulation of c-Myc (21). Because NAC can inhibit oridonin-induced apoptosis and senescence, the effect of NAC on the expression of these proteins was examined. As shown in Fig. 3A, the application of oridonin at 100 μM mainly led to apoptosis in SW1116 cells, as indicated by the degradation of PARP, which is a major substrate for caspases and a marker for apoptosis (27). Co-treatment with NAC inhibited the degradation of PARP. At 50 μM, oridonin mainly induced senescence in SW1116 cells, as reflected by the induction of p16, a hallmark of both replicative and accelerated senescence (28). In this case, co-treatment with NAC inhibited the induction of p16 (Fig. 3B).

The inactivation of c-Myc and the activation of p53 are important for both apoptosis and senescence (29–32). NAC co-treatment abrogated oridonin-induced downregualtion of c-Myc and upregulation of p53 in parallel with the inhibition of apoptosis and senescence (Fig. 3). These results indicated that c-Myc and p53 are involved in oridonin-induced senescence and apoptosis.

### Effects of catalase on ROS production, apoptosis and senescence in oridonin-treated SW1116 cells

**Figure 4:**
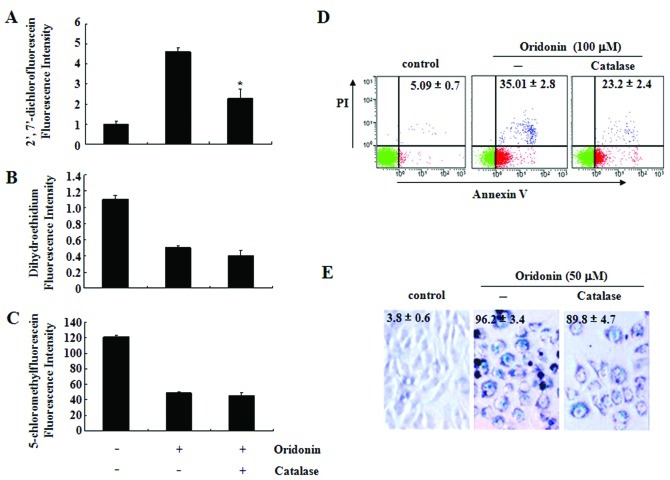
Effects of exogenous catalase on intracellular ROS production, glutathione depletion, oridonin-induced apoptosis and senescence in oridonin-treated SW1116 cells. SW1116 cells were treated with catalase and/or oridonin for 2 h. Intracellular (A) hydrogen peroxide, (B) superoxide anion, and (C) GSH levels were determined by FACS, respectively. The graphs show the mean fluorescence levels of (A) DCF, (B) DHE and (C) CMF, respectively. Cells were treated with catalase and/or oridonin (50 μM for 48 h or 100 μM for 12 h). (D) Annexin V-positive cells were measured with FACS. (E) Senescent cells were determined by senescence-associated β-galactosidase activity analysis (x100) . ^*^P<0.05 compared with the cells treated with oridonin alone.

To further determine the role of hydrogen peroxide in oridonin-induced apoptosis and senescence, SW1116 cells were treated with oridonin in the presence or absence of catalase (30 U/ml), a hydrogen peroxide-scavenging enzyme (33). Catalase partially reversed an oridonin-induced increase in hydrogen peroxide (Fig. 4A) but not the decrease of superoxide anion and GSH (Fig. 4B and C). Interestingly, catalase significantly decreased the percentage of Annexin V-positive cells (Fig. 4D) but not the senescence-associated β-galactosidase-positive cells induced by oridonin (Fig. 4E).

### Oridonin inhibits TrxR activity

**Figure 5:**
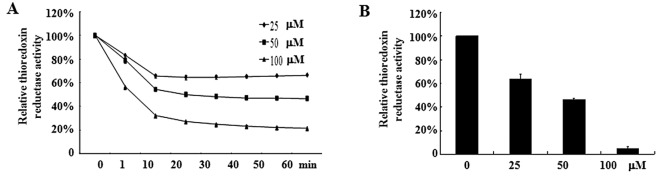
Inhibitory activity of oridonin on TrxR. (A) Recombinant rat TrxR (0.05 units) was incubated with various concentrations of oridonin for 1 h, and TrxR activity was measured by the dithionitrobenzene reduction assay. Error bars represent the standard deviations of duplicate experiments. (B) SW1116 cells were treated with different concentrations of oridonin for 2 h, and TrxR activity was determined. Error bars represent the standard deviations of duplicate experiments.

TrxR, an oxidoreductase that reduces oxidized proteins, represents an important valid target for cancer therapy. Because TrxR is easily targeted by electrophilic compounds and oridonin is a Michael acceptor with electrophilic propensity, we assumed that oridonin may directly inhibit TrxR activity, which causes an increase of hydrogen peroxide. As shown in Fig. 5A, oridonin caused a dose-dependent reduction of TrxR activity *in vitro*. Incubation of TrxR with 100 μM oridonin led to an 80% decrease in TrxR activity compared with DMSO treatment. In line with this result, SW1116 cells treated with oridonin at 50 or 100 μM for 2 h exhibited a marked decrease in TrxR activity (Fig. 5B). These results indicate that TrxR is one of the targets for oridonin.

## Discussion

We previously reported that oridonin induces potent growth inhibition, apoptosis, and senescence of colorectal cancer cells *in vitro* and *in vivo* (21). However, the underlying mechanism is largely unknown. In this study, we demonstrated that the antitumor activity of oridonin was attributed to its ROS increasing activity. Furthermore, TrxR inhibition is involved in this process.

ROS serve many cellular functions including actins as growth stimulants, second messengers, aging accelerants, cell death inducers and antibacterial agents. Based on our previous results and those of others (16,34), we hypothesized that oridonin may induce apoptosis and senescence in colon cancer cells via increasing ROS. Although it is currently not possible to precisely quantify the amount of subcellular ROS, tools to detect several kinds of ROS in the cells are available. With these chemical probes, we found that exposure to oridonin led to the increase of hydrogen peroxide and decrease of superoxide anions and GSH in SW116 cells and that the effects of oridonin on ROS level were dose-dependent. In the cellular environment, hydrogen peroxide can be derived from superoxide anions; this derivation can either occur spontaneously or can be catalyzed by superoxide dismutase (35). Whether oridonin could enhance the conversion of superoxide anions to hydrogen peroxide or inhibit the production of superoxide anions is currently unknown.

Many studies have demonstrated that hydrogen peroxide plays an important role in chemotherapy-induced apoptosis and senescence in cells (6–9). At low or mild concentrations, ROS seem to protect the cells or induce cell senescence, while at high concentration, ROS can initiate cell death by damaging many biological molecules. To reveal the role of hydrogen peroxide in oridonin-induced apoptosis, NAC, an ROS scavenger, were used in combination with oridonin. NAC completely abrogated the oridonin-induced increases of hydrogen peroxide levels and decreases of superoxide anion levels. Accordingly, oridonin induced apoptosis and senescence and the relevant changes of p53, p16 and c-Myc were also abrogated. These data indicate that hydrogen peroxide plays a key role in oridonin-induced apoptosis and senescence. Interestingly, when the levels of hydrogen peroxide were partially decreased by catalase, only the apoptosis but not the senescence was significantly reversed, indicating that the concentration of hydrogen peroxide determined the cell fate. These data suggest that hydrogen peroxide may play a major role in oridonin-induced apoptosis and senescence, although we cannot rule out tht other ROS, such as nitric oxide, or hydroxyl radicals may also be involved.

The mammalian thioredoxin system, which consists of NADPH, thioredoxin and thioredoxin reductase, plays an important role in protecting cells from oxidative stress-induced apoptosis (36). As overexpression of TrxR has been reported in several human cancers and is associated with decreased survival and resistance to chemotherapy, TrxR has been proposed as a target for anticancer drug development. Considering the extensive involvement of TrxR in the redox regulations and its close link with cancer and that it is easily attacked by electrophilic compound, we investigated the effect of oridonin on TrxR activity. Interestingly, oridonin dose-dependently inhibited the activity of TrxR *in vitro* and in cells. The trend was correlated to that of oridonin-induced hydrogen peroxide increase. Thus, oridonin may induce the accumulation of hydrogen peroxide by targeting TrxR.

In summary, we show here that hydrogen peroxide plays an important role in oridonin-induced apoptosis and senescence in colon cancer cells. We also provided evidence for the first time that oridonin could inhibit TrxR activity.
